# Pneumocystis jirovecii Pneumonia in Three Patients With Breast Cancer Receiving Neoadjuvant Dose-Dense Chemotherapy

**DOI:** 10.7759/cureus.21812

**Published:** 2022-02-01

**Authors:** Ritsuya Shiiba, Daisuke Himeji, Ryoichi Matsumoto, Gen-ichi Tanaka, Naoki Otomo

**Affiliations:** 1 Department of Internal Medicine, Miyazaki Prefectural Miyazaki Hospital, Miyazaki, JPN; 2 Department of Surgery, Miyazaki Prefectural Miyazaki Hospital, Miyazaki, JPN

**Keywords:** pneumocystis jirovecii pneumonia, lymphocytopenia, neoadjuvant chemotherapy, dose-dense chemotherapy, breast cancer

## Abstract

We report three cases of *Pneumocystis jirovecii* pneumonia (PJP) during dose-dense neoadjuvant chemotherapy for breast cancer. All patients presented with symptoms (e.g., fever), and computed tomography showed diffuse ground-glass shadows. Bronchoalveolar lavage was performed, and the diagnosis was confirmed by polymerase chain reaction for *Pneumocystis jirovecii*. All patients had completed three or four courses of dose-dense epirubicin-cyclophosphamide chemotherapy and received prednisolone for preventing chemo-induced nausea and vomiting. Moreover, lymphocytopenia was observed in all patients. Since the onset of PJP in preoperative neoadjuvant chemotherapy can be life-threatening and leads to delayed surgery, careful consideration of prophylaxis for PJP is required.

## Introduction

*Pneumocystis jirovecii *pneumonia (PJP) can be a life-threatening opportunistic infection in immunocompromised hosts. Because of improvements in human immunodeficiency virus (HIV) treatments, and as more patients receive immunosuppressive therapy, PJP has become common in non-HIV-infected cases, including solid malignancies such as lung and breast cancer [1].

Dose-dense chemotherapy, which shortens dosing intervals and increases dose intensity, is recommended as a perioperative breast cancer treatment. It has been reported to reduce the risk of recurrence and mortality by reducing the usual three-week dosing interval to two weeks [2-3]. Recently, pegfilgrastim, a long-acting granulocyte colony-stimulating factor (G-CSF) preparation [2], has been approved and dose-dense therapy became widely used in Japan. However, there are several reports of PJP onset during dose-dense therapy, and Waks et al. identified 19 cases of PJP among a series of 2,057 patients who received the dose-dense adriamycin and cyclophosphamide (AC) regimen for early breast cancer and reported an overall incidence of 0.6% (95% confidence interval 0.3-1.0%) [4]. Herein we report three cases of PJP during neoadjuvant chemotherapy (NAC) for breast cancer.

## Case presentation

Case 1

**Figure 1:**
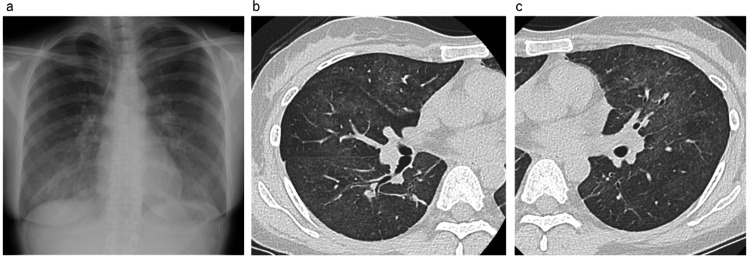
Chest radiography and high-resolution computed tomography (HRCT) of Case 1 on admission a: Chest radiography showing bilateral diffuse ground-glass opacity; b, c: HRCT showing bilateral diffuse ground-glass opacities with a subpleural sparing pattern

A 40-year-old Japanese woman diagnosed with stage ⅢC left breast cancer had received a neoadjuvant dose-dense (dd) epirubicin-cyclophosphamide (EC) regimen with pegfilgrastim support (consisting of epirubicin hydrochloride (90 mg/m^2^), cyclophosphamide hydrate (600 mg/m^2^), and dexamethasone 6.6 mg on Day 1; pegfilgrastim on Day 2; and dexamethasone 8 mg on Days 2-4). Chemotherapy was administered every two weeks for four cycles of treatment. After this, she was treated with weekly paclitaxel (80 mg/m^2^). Seven days after paclitaxel treatment, she was admitted to our department owing to fever and dyspnea. Her vital signs were as follows: temperature 38.6°C; blood pressure 109/86 mmHg; pulse 110 beats/min; and respiratory rate 20 cycles/min with an O_2_ saturation of 96% on room air. No anomalies were noted on auscultation. Laboratory results revealed normal white blood cell count, slightly decreased lymphocyte counts, and a normal C-reactive protein (CRP) level. Krebs von den Lungen (KL)-6 and surfactant protein D (SP-D) were elevated. β-D-glucan levels were measured with a kinetic turbidimetric assay by employing the WAKO^TM^ Beta-Glucan test (Wako Pure Chemical Industries, Tokyo, Japan). The serum β-D-glucan level was normal (Table 1). Additionally, renal and liver function tests showed normal results. Chest radiograph showed ground-glass opacity of the bilateral lung field. Chest computed tomography (CT) also revealed bilateral ground-glass opacities (Figure 1).

**Table 1:** Laboratory findings of breast cancer patients diagnosed with PJP PJP: *Pneumocystis jiroveci* pneumonia, WBC: white blood cell, LDH: lactate dehydrogenase, CRP: C-reactive protein, KL-6: Krebs von den Lungen-6, SP-D: surfactant protein D

Case	WBC (cells/mm^3^)	Lymphocyte (cells/mm^3^)	LDH (U/L)	CRP (mg/dL)	KL-6 (U/mL)	SP-D (ng/mL)	β-D glucan (pg/mL)
Reference ranges	3,300-8,600	545-4257	106-220	0.00-0.30	0-500	<110	0.0-10.0
1	5,580	1,127	635	0.05	671	257	4.3
2	2,400	120	195	5.73	345	51.7	<2.8
3	9,090	680	307	1.00	544	173	<3.0

Based on these findings, we suspected PJP or drug-induced pneumonia. Since her respiratory condition was stable, we decided on treatment with trimethoprim/sulfamethoxazole (TMP 15 mg/kg/day / SMX 75 mg/kg/day) without using systemic corticosteroids. Due to a scheduling problem in the endoscopy room, we performed bronchoalveolar lavage (BAL) on the sixth day after admission. An analysis of the BAL fluid (BALF) revealed a total cell count of 2.19 × 10^6^/mL (neutrophils 6%, lymphocytes 77%, histiocytes 16%, and eosinophils 1%) with no malignant cells or pathogenic organisms detected. Her clinical condition improved with these treatments. One week after bronchoscopy and BAL, polymerase chain reaction (PCR) of her BALF sample was positive for *Pneumocystis jirovecii*. Based on these results, she was diagnosed with PJP and we continued TMP/SMX treatment. Treatment with TMP/SMX continued for 14 days. After these treatments, her general condition and CT findings improved, and PJP prophylaxis was also initiated with TMP/SMX. Subsequently, weekly paclitaxel therapy was resumed and neoadjuvant chemotherapy was continued with prophylactic TMP/SMX. The operation was performed without any complications.

Case 2

**Figure 2:**
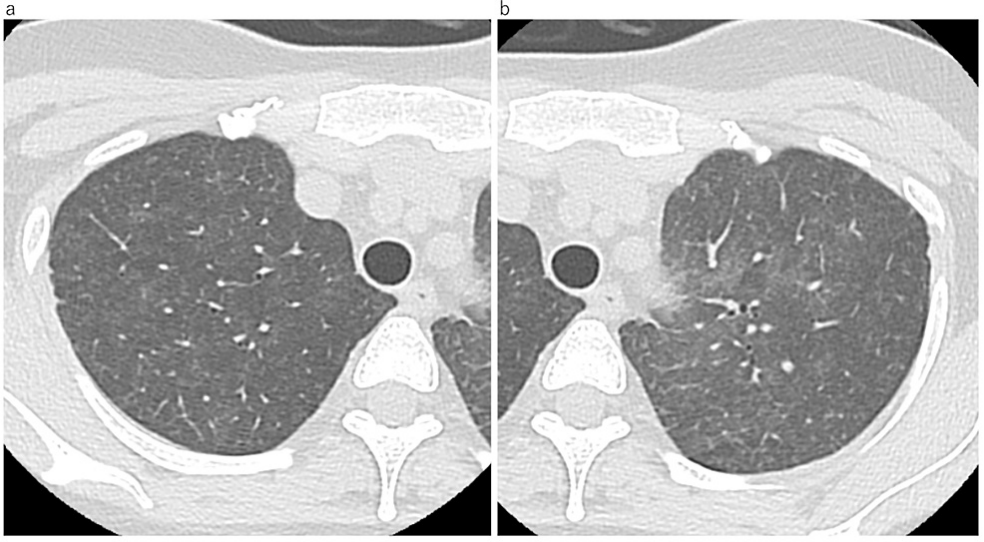
High-resolution computed tomography (HRCT) of Case 2 on admission a,b: HRCT showing slight bilateral diffuse ground-glass opacity.

A 46-year-old Japanese woman diagnosed with stage II B right breast cancer had received a neoadjuvant dose-dense EC regimen with pegfilgrastim support. After the end of the fourth course of chemotherapy, she visited our hospital complaining of fever. Her vital signs were as follows: temperature 37.6°C; blood pressure 109/76 mmHg; pulse 111 beats/min; and respiratory rate 24 cycles/min, with an O_2_ saturation of 96% on room air. No anomalies were noted on auscultation. Laboratory tests revealed a decreased white blood cell count and lymphocyte count, elevated CRP level, and normal KL-6 and SP-D levels (Table 1). The serum β-D-glucan level was normal. Her chest radiograph showed ground-glass opacity of the bilateral lung field. Chest CT also revealed bilateral ground-glass opacities (Figure 2).

As in the first case, she underwent bronchoscopy and BAL. An analysis of the BALF revealed a total cell count of 2.48 × 10^5^/mL (cell fractionation of bronchial lavage fluid unknown) with no malignant cells or pathogenic organisms detected. Drug-induced pneumonia can often be severe, and at this point, the possibility could not be ruled out and her respiratory condition worsened. Thus, we treated her with systemic corticosteroid therapy with oral prednisolone at 80 mg/day and TMP/SMX (TMP 15 mg/kg/day / SMX 75 mg/kg/day). One week after bronchoscopy and BAL, PCR of her BALF specimen was positive for *Pneumocystis jirovecii* DNA. Based on these results, she was diagnosed with PJP, and we continued corticosteroid and TMP/SMX treatment. Prednisolone was gradually tapered off over three weeks. TMP/SMX continued for 14 days. After these treatments, her general condition improved. Subsequently, 12 courses of weekly paclitaxel therapy were performed with prophylactic TMP/SMX, neoadjuvant chemotherapy was completed without relapse of pneumonia, and surgery was performed as scheduled.

Case 3

**Figure 3:**
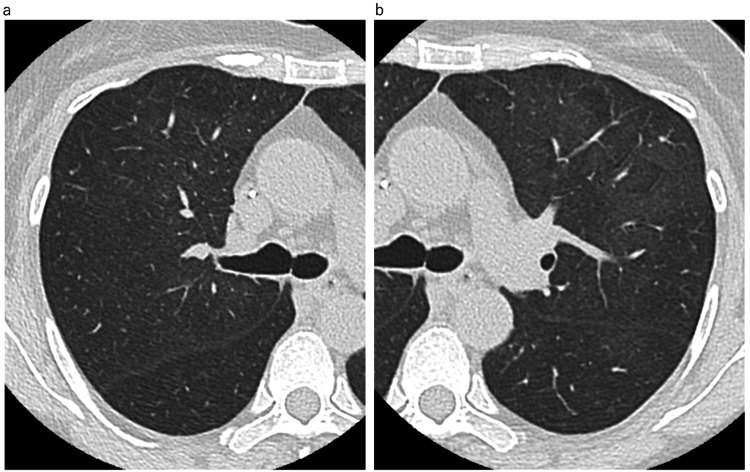
High-resolution computed tomography (HRCT) of Case 3 on admission a,b: HRCT showing slight bilateral diffuse ground-glass opacity.

A 57-year-old Japanese woman diagnosed with stage II B right breast cancer had received the same neoadjuvant dose-dense EC regimen with pegfilgrastim support. After three courses of EC therapy, she was referred to our department after complaining of fever. Her vital signs were as follows: pulse 88 beats/min and O_2_ saturation of 98% on room air. No anomalies were noted upon auscultation. Laboratory tests revealed an elevated white blood cell count, decreased lymphocyte count, and a slightly elevated CRP level (Table 1). Her KL-6 and SP-D levels were slightly elevated. The serum β-D-glucan level was normal (Table 1). Her chest radiograph showed ground-glass opacity of the bilateral lung field. Chest CT also revealed bilateral ground-glass opacities (Figure 3).

As in the first case, she underwent bronchoscopy and BAL. An analysis of the BALF revealed a total cell count of 9.84× 10^5^/mL (neutrophils 10%, lymphocytes 69%, histiocytes 18%, and eosinophils 3%) with no malignant cells or pathogenic organisms detected. Since her respiratory condition did not worsen, we decided on treatment with TMP/SMX (TMP 15 mg/kg/day / SMX 75 mg/kg/day) without systemic corticosteroid therapy. One week after bronchoscopy and BAL, PCR of her BALF sample was positive for *Pneumocystis jirovecii* DNA. Based on these results, she was diagnosed with PJP, and we continued TMP/SMX treatment. Treatment with TMP/SMX continued for 14 days. After these treatments, her general condition and chest radiograph and CT findings improved. Following that, one course of ddEC therapy was performed, and then 12 courses of weekly paclitaxel therapy were performed with prophylactic TMP/SMX. During the course, there was no recurrence of pneumonia, neoadjuvant chemotherapy could be continued, and surgery was performed as scheduled.

## Discussion

This is the first case series of PJP that developed during dose-dense neoadjuvant chemotherapy for breast cancer in Japan. Dose-dense chemotherapy, which shortens the dosing interval and increases dose intensity, is recommended as perioperative treatment for patients with breast cancer. Dose-dense AC and EC therapies have been reported to reduce the risk of recurrence and mortality by reducing the usual three-week dosing interval to two weeks [3,5]. In order to administer dose-dense therapy, it is necessary to administer a granulocyte-colony stimulating factor (G-CSF) preparation prophylactically. With the approval of pegfilgrastim, a long-acting G-CSF preparation, in September 2014, dose-dense therapy became widely used in Japan. Along with this, there are isolated case reports and one case series of PJP during dose-dense neoadjuvant chemotherapy for breast cancer [4,6-7].

In the presented cases, high-resolution CT scans of the patients revealed a diffuse ground-glass opacity with patchy distribution and subpleural sparing pattern. Although these imaging findings are common in PJP [8], they are also found in interstitial lung disease (ILD) such as drug-induced ILD. However, in patients with breast cancer receiving neoadjuvant chemotherapy (NAC) or adjuvant chemotherapies, ILD is considered a rare adverse effect. Few studies have reported the frequency of ILD in detail, and only a few cases have been described in the literature [9]. In pivotal phase III clinical trials, with standard NAC or adjuvant chemotherapies, such as dose-dense EC therapy [2-3], AC [10], or AC followed by paclitaxel or docetaxel [11], no ILDs were reported as adverse events, with the exception of one case of pneumonia in a patient who received EC [12]. In fact, in Case 1 and Case 3, pneumonia was improved by TMP/SMX therapy alone without systemic corticosteroid therapy, and in Cases 1 and 3, the same regimen of paclitaxel or ddEC therapy with TMP/SMX prophylaxis after pneumonia treatment was administered without pneumonia flare-up. This clinical course suggests that the pneumonia in Case 1 and Case 3 is different from that of drug-induced interstitial pneumonia. In Case 2, the patient was also treated with systemic steroids, and the anticancer agents used in the preoperative chemotherapy were changed; hence, drug-induced interstitial pneumonia cannot be completely ruled out from this course of treatment. In addition, these patients were considered to be at a high risk of developing PJP, we performed BAL for PJP diagnosis. The PCR assay of these patients’ BALF specimen was positive for *Pneumocystis jirovecii*. In general, microscopic detection of *Pneumocystis jirovecii* in respiratory specimens has been the gold standard for the diagnosis of PJP [13]. However, non-HIV-infected patients with PJP have a lower burden of pneumocystis than HIV-positive patients, and the organisms are difficult to detect via microscopic examination [13]. In contrast, PCR assay has 85%-100% sensitivity and 79%-96% specificity for the diagnosis of microscopically positive PJP [14-15]. Thus, a PCR assay is increasingly being used for the microbiological diagnosis of PJP.

Previous studies had demonstrated that the serum β-D-glucan assay can be useful for the diagnosis of PJP [15]. The serum β-D-glucan levels of our patients were low (4.3 pg/ml, <2.8 pg/ml, and <3.0 pg/ml for the three patients). Li et al. reported that the sensitivity of β-D glucan in non-HIV patients was relatively low compared with that in HIV-positive patients and, based on their meta-analysis, concluded that the decreased positivity rates might be explained by the lower burden of Pneumocystis in non-HIV PJP patients [16]. Therefore, the low β-D glucan level in our cases does not deny the possibility of PJP and is considered to reflect the low pneumocystis burden. In addition, some reports have defined Pneumocystis-colonization (false positive) as *Pneumocystis*​​​​​​​* jirovecii*-PCR positive in respiratory samples from patients without clinical features of PJP or CT findings [17]. Our cases had clinical features of PJP, characteristic imaging findings, and positive results of PCR for *Pneumocystis​​​​​​​ jirovecii*; therefore, we diagnosed our patients with PJP.

As described above, Waks et al. identified 19 cases of PJP among a series of 2,057 patients who received the dose-dense AC regimen for early breast cancer and reported an overall incidence of 0.6% (95% confidence interval 0.3-1.0%) [4]. In our institute, we identified three cases (3.6%) of PJP in 83 patients receiving chemotherapy with the dose-dense EC-containing regimen (Table 2). These reports do not enable simple comparisons owing to the small number of cases but suggest that there may be racial differences in the risk of developing PJP with dose-dense chemotherapy. It is necessary to examine the incidence of PJP in a larger study.

**Table 2:** Breast cancer patients characteristics diagnosed with PJP EC: epirubicin hydrochloride + cyclophosphamide hydrate; PTX: paclitaxel; PJP: *Pneumocystis jirovecii* pneumonia

Case	Age	Chemotherapy regimen before PJP diagnosis	Days from the start of chemotherapy to PJP diagnosis	Average prednisone equivalent day (mg) from chemotherapy start to PJP diagnosis	The lowest lymphocyte count around PJP diagnosis
1	42	Dose-dense EC×4 +weekly PTX×1	76	11.35mg	217 /μL
2	46	Dose-dense EC×4	59	13.69mg	140 /μL
3	57	Dose-dense EC×3	51	11.88mg	450 /μL

There is an interesting commonality about the onset of PJP in patients with breast cancer treated with dose-dense neoadjuvant chemotherapy. Waks et al. reported that all patients with PJP were diagnosed after three or four cycles of dose-dense AC chemotherapy. In the other two cases, PJP developed after four cycles of dose-dense AC chemotherapy. Our three cases also developed PJP after completing three or four courses of dose-dense EC chemotherapy.

First, it is necessary to consider the possibility that the dose and duration of steroid treatment pose a risk for developing PJP. In these cases, steroids are mainly administered to prevent chemotherapy-induced nausea and vomiting (CINV). Waks et al. reported that the average dose of corticosteroids was 16 mg prednisone equivalent per day over the course of their approximately 56-day-long AC chemotherapy course [4]. The other two cases also received 11 and 19 mg prednisone equivalent per day. Three patients in our study received 40 mg dexamethasone every two weeks as CINV prophylaxis or 12.3 mg prednisone equivalent per day (Table 2). As a criterion for PJP prophylaxis for patients without HIV, the risk of developing PJP has been reported when 20 mg prednisolone-equivalent steroid is taken for ≥1 month [18]. In our cases, although the amount of prednisolone was relatively low, corticosteroids were administered for approximately two months. We think the inclusion of corticosteroid therapy in the chemotherapy regimen will be a risk factor for developing PJP. Hence, caution was required.

Another possible risk factor is the number of lymphocytes. In patients without HIV, lymphocytopenia has been suggested to be associated with the risk of developing PJP in SLE [19], but this is controversial in breast cancer chemotherapy. All of our patients also showed Grades 3-4 lymphocytopenia. Therefore, lymphocyte counts should be monitored during dose-dense chemotherapy, and prophylaxis may be considered when severe lymphopenia is observed.

Finally, the indication of prophylaxis consideration should be discussed. Green et al. described that PJP prophylaxis in the non-HIV population is warranted when the risk for PJP is estimated to be >3.5% [20]. As mentioned above, the data of our hospital suggest that prophylaxis is appropriate, but the frequency of PJP onset needs further study. At a minimum, if lymphocytes decrease during the course, it is necessary to consider prophylaxis for preventing PJP.

## Conclusions

In conclusion, this report is the first case series of PJP developing during neoadjuvant treatment for breast cancer with dose-dense chemotherapy in Japan. Since the onset of PJP in preoperative neoadjuvant chemotherapy can be life-threatening and leads to delayed surgery, careful consideration of prophylaxis for PJP is required.
